# Evaluation of Good Nursing Practices for women in situations of domestic violence*

**DOI:** 10.1590/1980-220X-REEUSP-2025-0374en

**Published:** 2026-01-23

**Authors:** Mariana Sbeghen Menegatti, Rosa Maria Godoy Serpa da Fonseca

**Affiliations:** 1 Universidade de São Paulo, Escola de Enfermagem, São Paulo, SP, Brazil.

**Keywords:** Nursing, Evidence-Based Nursing, Primary Health Care, Quality Indicators, Health Care, Domestic Violence, Public Health

## Abstract

**Objective:**

To identify indicators used to evaluate Good Nursing Practices developed in Primary Health Care for women in situations of domestic violence.

**Method:**

A qualitative study conducted with primary care nurses in a Brazilian municipality. Semi-structured interviews were developed, and the Software *WebQDA* was used for data analysis. The analytical categories used were: gender violence and nursing care according to the Theory of Nursing Praxis Intervention in Collective Health.

**Results:**

Empirical categories emerged: 1) Conception of service evaluation by service users; 2) Evaluation practices: absence of a formal evaluation structure and professional self-evaluation; 3) Potential and used indicators for evaluating care.

**Conclusion:**

Two indicators already in use were identified, and others were proposed, highlighting the potential for building collaborative instruments. Despite the existence of protocols and policies, an evaluation tool is required, which can benefit from the theoretical and methodological guidance of the Theory of Nursing Praxis in Collective Health.

## INTRODUCTION

Good Nursing Practices (GNP) in Primary Health Care (PHC) are characterized by the development of interventions within the geopolitical territory of social production and reproduction of individuals and communities. The professionals who carry them out aim to understand and transform the health-disease profiles of the social groups present there. By prioritizing actions based on the perspective of the social determinants of health, their aim is to impact the dimensions of objective reality: singular (of individuals), particular (of social groups), and structural (which corresponds to the broader spheres of society)^(1)^.

When employed to combat domestic violence in the territories, the GNP strategies are structured around actions guided by the epidemiological profile of the social groups present in the territory, including the recognition of their lifestyles and work patterns, as well as the phenomena that make women vulnerable to violence. A critical understanding and interpretation of the social production and reproduction experienced by women is essential for recognizing how violence is determined, manifested, and reproduced^(2)^.

Professionals working in primary health care recognize the importance of their role and highlight the need for guidelines and a clear definition of roles, as well as evidence-based guidance, for the care of women experiencing violence, considering the context in which they live. The structuring of the interventions evidenced is based on constant critical reflection, using protocols and guidelines appropriate to the local reality and monitoring results through evaluations^(3)^.

In PHC, assessment is a form of guidance that contributes to decision-making and the (re)direction of actions aimed at transforming the population’s life and health reality^(4)^. The evaluation of health services and interventions in general has been the subject of research, and it is noteworthy that studies focused on evaluating interventions from a Collective Health perspective are less common, and the evaluation of GNP aimed at women experiencing violence is not a specific focus of attention^(5,6,7)^.

Considering the knowledge gap related to instruments for evaluating interventions from a Collective Health perspective, it is understood as important to develop research focused on recognizing indicators or instruments for evaluating assistance to women in situations of domestic violence.

Indicators are parameters, either qualitative or quantitative, used to analyze the processes and results of a given action. Thus, they are important management tools that allow monitoring situations that should be changed, encouraged, or enhanced^(5,8)^.

Identifying and analyzing how nurses evaluate the care they provide is important for understanding what guides their professional practice and the outcomes of that care. Therefore, the objective of this study was to identify indicators used to evaluate the GNPs developed in Primary Health Care for women experiencing domestic violence.

## METHOD

### Study Design

This was a qualitative study that, aimed at ensuring quality and reliability, was carried out according to the guidelines of the tool *Consolidated Criteria for Reporting Qualitative Research* (COREQ). This article is the product of a master’s thesis defense in the Nursing Graduate Program at the Nursing School of the Universidade de São Paulo^(9)^.

### Location and Period

The research was conducted in the municipality of Araraquara, SP, Brazil, with data collection between June 3 and July 15, 2022.

### Population and Selection Criteria

The target population consisted of nurses working in PHC for at least six months. The selection of participants was done through snowball sampling. Initially, two seeds were randomly selected from the list of professionals who were part of the PHC teams in the municipality, and from then on, the other participants were indicated by the previous ones.

### Data Collection

For data collection, a questionnaire consisting of questions characterizing the participants in relation to their education and work. Subsequently, an in-depth interview was conducted following a semi-structured script that involved knowledge and experiences related to GNPs in PHC for women in situations of domestic violence, in addition to inquiring about the evaluation strategies employed.

Both the characterization questionnaire and the semi-structured script were tested in a pilot study with a PHC professional from the municipality, who was part of the authors’ research group’s contact network and who was excluded from the sample. The test allowed for adjustments to the instrument and adaptation for the development of the proposed data collection.

Before sending out invitations to participate in the research, and with the support of the Health Department of Araraquara, a presentation was held about the researchers, the research group, the project, and the objectives of the work to be developed. At the event, professionals were given the opportunity to ask the researchers questions, and the freedom of participation in the interviews was emphasized, while reiterating the anonymity of those who agreed.

Subsequently, the nurses were contacted by telephone and invited individually to participate. It should be noted that the semi-structured interviews were conducted by telephone, given the context of the COVID-19 pandemic. The professional who expressed interest in participating received an email containing a link to access the Google Forms to complete the Informed Consent Form and chose a date and time for the interview, being instructed to choose a private, quiet environment, free from interruptions and in which they felt comfortable to conduct the interview. The interviews lasted between 56 minutes and 1 hour and 42 minutes. All the professionals contacted agreed, and none dropped out during the data collection process.

The interviews were conducted by the first author, a nurse, who at the time was a graduate student in a master’s program in Collective Health Nursing. The supervision of data collection and other steps was carried out by the second author, a nurse with a doctorate degree, associate professor, and advisor in the aforementioned graduate program. Both authors have extensive experience in qualitative research, including semi-structured interviews among other data collection methods.

Data collection continued until data saturation was reached, which occurred with the 12th interview. Following this, the authors discussed the findings and agreed to conduct three more interviews to confirm saturation, bringing the total number of professionals interviewed to 15.

### Data Analysis and Treatment

The information was stored on an electronic device accessed exclusively by the researchers, excluding any records stored in the cloud, thus guaranteeing the confidentiality of the information. The participants were identified as E1, E2, ...E15, ensuring anonymity. The characterization data were extracted from Google Forms into an Excel spreadsheet and underwent descriptive analysis.

The audio recordings of the interviews were transcribed and saved in *Portable Document Format* (pdf) and submitted to qualitative analysis, through the Thematic Content Analysis technique, consisting of the following stages: 1) pre-analysis, 2) exploration of the material, 3) processing, interpretation of results, and inference^(10)^. Support was provided by Software *Web Qualitative Data Analysis* (WEBQDA), available in an online environment, which through the coding system allowed codes to emerge that were refined and grouped, giving rise to the coding tree^(11)^ from which the empirical categories presented in the results emerged.

The results were discussed using the analytical categories: gender violence and nursing care according to the Theory of Nursing Praxis Intervention in Collective Health (TIPESC)^(12)^.

Gender-based violence is expressed in a set of violent acts perpetrated against a person because of their gender, with women being the predominant victims of this violence, and these acts operating both explicitly and symbolically. Violence against women is one expression of gender-based violence, given the inequality imposed between men and women and the sociocultural norms that grant greater power to men over women, including in relationships that occur within family spaces or in cohabitation^(13)^.

Nursing care according to TIPESC is guided by the worldview of historical and dialectical materialism, which understands the health-disease process as socially determined. TIPESC’s philosophical foundations are historicity and dynamism, and its theoretical foundations are conceptual categories (comprehensive sets of historically constructed notions and ideas that demarcate the interconnected parts of the phenomenon under consideration in its spaces) and dimensional categories (praxis, totality, interdependence between the structural, the particular, and the singular)^(12)^.

### Ethical Aspects

Resolution 466, of December 12, 2012, of the National Health Council was followed^(14)^, along with the guidelines for research in a virtual environment, from the National Research Ethics Committee^(15)^. Data collection occurred with the consent of the participants in the Informed Consent Form. The project was submitted to the Research Ethics Committee of the School of Nursing at the Universidade de São Paulo and authorized on February 25, 2022, CAAE: 52878021.3.0000.5392, Opinion 5.266.104.

## RESULTS

Of the 15 participants, nine worked in Family Health Units and six in other PHC services, with lengths of service ranging from 7 months to 35 years, with an average of 13.9 years. All reported providing care to women experiencing violence throughout their work in PHC. Fourteen people were women and one was a man; eleven were married, two divorced, one in a common-law marriage, and one single; all had children.

Regarding the year of graduation, the oldest was 1977 and the most recent 2020, with 60% between 1991 and 2010. Fourteen professionals stated that they have completed or are currently pursuing a graduate certificate. Regarding graduation, three of them had completed a master’s degree. Seven professionals had already completed some training on GNP in PHC, with topics related to immunization, humanization, violence against women, embracement, violence against children and adolescents, care for vulnerable populations, homeless populations, COVID-19, semiology, breastfeeding, the National Primary Care Policy, the responsibilities of nurses, and protocol-based care.

From the qualitative analysis of the data, the following empirical categories emerged: 1) The conception of service evaluation by service users; 2) Evaluation practices: the absence of a formal evaluation structure and professional self-evaluation; 3) Indicators used and potential indicators for evaluating care.

### The Concept of Service User Evaluation

The first empirical category reveals cognitive and operational obstacles in implementing the evaluation of the care received by users. The professionals had doubts about conducting any evaluation of the care provided by the women attended at the service. They believed that women lacked knowledge about evaluating the care they received.

*I think they don’t evaluate, I think they don’t have that knowledge.* (E7)

The possibility of evaluating service through the municipality’s ombudsman emerged, but this is not a practice carried out by all municipalities.

*They can do it through the ombudsman’s office, but not everyone does.* (E15)

One response that the nurses considered an evaluation of the user was the woman’s behavior during the care process, the bond formed, and the demonstration of trust in the team.

*I think it’s from the feedback regarding bond, of coming, telling us what happened. So, saying they’re grateful, no, not that, but like... comes, trusts, tells what happened, what she did, that she’s okay now.* (E5)

*Assessment practices: the absence of a formal assessment structure and professional self-assessment.*

The second category includes the forms of assessment that were being used, even if in a non-systematic way.

The lack of a formal post-service evaluation, as well as evaluation instruments, was highlighted.

*There is no post-service evaluation of what could have been done better. This assessment, of saying... Ah, that service, we did that... Could we have done better? I’ve never done that at my job. At least in terms of violence.* (E9)

*No, I don’t conduct evaluation processes. I provide the service, I monitor the service, but I have no way of evaluating it.* (E8)

Despite denying the existence of formal assessment instruments, the participants highlighted the performance of self-assessments, in an empirical way.

*If there is an evaluation, it’s more of a self-evaluation (...) where I often question whether I’m doing what I should be doing.* (E7)

*It’s empirical; it’s done through observation.* (E5)

The interviewer noted that the questions related to evaluation caused discomfort among the professionals, who needed some time to reflect on what they understood as an evaluation action for their professional practice. There was also concern about acknowledging that no evaluation strategies were being employed.

### Indicators Used and Potential for Evaluating Care

The last category maps existing and suggested instruments. One interviewee stated that there is an annual evaluation of care by the PHC management, where nurses discuss the topic together with the municipality’s continuing education team, considering the current situation and any potential shortcomings.

*I know there’s a conversation that’s been going on for a long time. This protocol has been improving, and I know that there is indeed a periodic evaluation of the actions. [...] There they give us a picture of the municipality, what the forms of violence are, where we might be failing, and what we can improve.* (E10)

The nurses cited the number of notifications as an indicator used to measure care, with the possibility of linking it to monitoring what was done.

*You see, we have a notification of violence [...] we feed it into the system and then we get this violence indicator. We document all the steps taken, everything we did, to determine the appropriate course of action...* (E12)

An indicator of women’s social vulnerability, involving education, income, family structure, and comorbidities, was identified as both useful and essential.

*[...] one that would be essential is the social vulnerability indicator. We work with some vulnerability scales. We work within the family context. So, I believe that education level, income, family structure, and comorbidities are things that we’ve seen, as they affect a person more, especially me, working in a very peripheral region, there is a social impact that ends up being reflected in health actions.* (E6)

One participant highlighted the need for an indicator that assesses the uptake of cases by Primary Care, considering the service’s connection with the community.

*So, given its connection to the community, AB would need an indicator that reflects the detection of these cases, and I can’t yet figure out how we would evaluate that.* (E10)

Indicators were suggested to evaluate women’s experience with healthcare, such as the embracement, difficulties encountered, and what was relevant during the service.

*I think it would be exactly what she suffered. How our service helped her [...] what the worst difficulty she had was.* (E4)

*Hmm, I think that perhaps a conversation with her, an interview, even through some instrument that she could use to show whether she managed to get out of this situation or whether she didn’t manage to get out [...]* (E14)

Teamwork was suggested, although it was considered subjective and difficult to implement. Another suggestion was a referral indicator for other services within the network.

*Look, indicator... It would be a more subjective matter. Because that would be, for example, an evaluation of teamwork [...].* (E11)

*Perhaps referrals to reference services, protection services...* (E3)

Indicators of adherence to care and outcome were also suggested, including: overcoming or not overcoming the violence, regaining independence, employment, return to violence, abandonment of follow-up, or discharge.

*I think the main indicator for us to achieve this would be adherence itself...* (E12)

*I think it’s important for us to know how effective the service is. So, if it was effective, how many did you manage to remove from violence, and how many returned to a state of violence?* (E9)

*The dropout rate I have is a concrete thing [...]. How many closed the follow-up[...], and then, when I close, I close as discharge....* (E8)

The possibility of suggesting indicators that they considered important and applicable seemed to generate comfort among the professionals, according to the interviewer’s observations. Noticeably, at this point, the interview became more fluid, possibly because the suggestions were an action to overcome the discomfort that emerged from the recognition that there was no systematized evaluation.

Furthermore, regarding the records made by the researcher, a difference was identified in the interviews of the female participants compared to the male participant. The male nurse’s interview lasted 56 minutes, compared to the female nurses’ interviews, which always lasted more than 1 hour and 16 minutes. There was a noticeable increase in involvement, including emotional engagement, and concern about the topic among the women interviewed when discussing the assistance provided.

Based on the responses, 11 suggested indicators were compiled, two of which were already in use and nine others that were suggested, as shown in Chart 1.

**Chart 1:** Indicators used and suggested for evaluating Good Nursing Practices aimed at women experiencing violence in Primary Health Care – São Paulo, SP, Brasil, 2025.

Indicators
Number of reported cases of domestic violence
Number of cases of violence identified in Primary Health Care.
Number of referrals made
Number of women served by the network’s services referred by Primary Health Care.
Adherence to care, guidelines
Analysis of the woman’s experience (support or difficulties faced)
Teamwork (professionals who attended to and intervened in the situation experienced by the woman)
Analysis of vulnerabilities: education, income, family composition and dynamics.
Rate of return to the service(s)
Time required to resolve the situation currently being addressed.
Case outcome: abandonment or discharge

## DISCUSSION

The characterization of the participants shows that most of them have extensive experience in primary health care, which can facilitate the identification and management of complex situations, such as violence against women. The predominance of women reflects the predominantly female composition of nursing, often associated with caregiving and supportive practices. The diversity in terms of training and professional experience suggests the coexistence of different professional generations, which can broaden perspectives on the subject. The number of professionals with a graduate certificate and the presence of masters indicates investment in continuing education, while participation in courses on GNP, including topics related to violence against women, demonstrates a search for qualifications to care for women.

The professionals interviewed made assumptions regarding whether the women they served were conducting assessments, even suggesting that they did not perform them and that the women lacked the knowledge to do so.

Women’s knowledge encompasses, beyond their level of education, an understanding of the right to health, the quality of services, and the mechanisms of accountability and effectiveness, which can be influenced by the social group to which these women belong and the community in which they live^(16)^. In other words, both the impact of the actions taken and their criticism are directly influenced by the social context in which they are carried out^(17)^.

For example, the evaluation of a service may encounter interference when the women being served lack knowledge about their rights within the health system, or when there are issues such as embracement or privacy within the service. Thus, a woman may not know that there is the possibility of expressing praise, criticism, or suggestions, or she may understand that what was offered to her was the best that could be done, and this “best” may, in reality, have fallen short of what is stipulated in policies or protocols.

Tools or strategies involving the community with the aim of strengthening its relationship with professionals facilitate and promote the creation of opportunities to identify obstacles or solutions to improve the care provided within health services, which is a fundamental part of ensuring the user’s social responsibility^(16)^.

Among the findings, some professionals highlighted the feasibility of women evaluating the care they receive through institutional channels, such as the municipality’s ombudsman. This is a democratizing constitutional device that allows for direct input from the population and has the functions of giving individuals an active voice, allowing for criticism, suggestions or complaints, enabling the execution of social control through oversight, and evaluating user satisfaction^(18)^. It is worth questioning its dissemination and encouraging services to publicize the existence of the ombudsman’s office and its purpose, as well as access to the channel through different strategies, avoiding means that limit the reach of certain social groups, such as exclusive access through online pages, social networks, or telephone.

The nurses described one way in which they recognize a woman’s evaluation of the care provided: through the woman’s interaction with the team and her expression of gratitude. A study dedicated to analyzing care indicators states that adherence to the therapeutic-care plan and increased trust in professionals can be considered for evaluating GNP^(5)^.

In line with the uncertainty surrounding the evaluation by women and their experience with the services, the professionals suggested, and the third empirical category emerges, an evaluation of women’s experiences while receiving care. The experience before, during, and after care, as well as how and when the person uses and needs the health service, are aspects that directly affect satisfaction. Despite the influence that experience has on satisfaction, it is important to highlight that satisfaction is a relative concept, influenced by aspects such as the person’s expectations and perception of the services received and the professionals who served them^(19)^. A person can be satisfied even without having had a good experience, which may lead them to not return to the service^(20)^.

User experience received attention from the Brazilian Ministry of Health in an Ordinance that provides for indicators for performance-based payment in Primary Health Care (APS) for the years 2021 and 2022, including indicators for evaluating the quality of care and patient experience^(21)^. The Self-Assessment Manual for Improving Access and Quality (AMAQ), reflective and problem-solving in nature, includes in the evaluation of the healthcare process, in addition to professionals and managers, the service user in terms of participation, social control, and satisfaction^(22)^. However, this research did not mention any of the strategies or evaluation items proposed in the cited documents.

Regarding the implementation of management evaluations of GNP, doubts arose concerning a formalized execution. Thus, this corroborates what the literature highlights as a gap in the implementation of evaluations of healthcare professionals’ practices. Evaluation remains a challenge for most professionals in the field, given the complexity of the subject^(5)^.

The use of empirical self-assessments by professionals was highlighted. Since the self-assessment mentioned does not follow guiding principles, it becomes a reflective process for the professional regarding their performance. It is important that the self-assessment process be formalized and directed, so that professionals perceive themselves within a safe process of change that involves movement, listening, and consideration of possibilities that will contribute to the planning of future actions, pointing to new reasoning and new solutions^(23)^.

A nurse stated that there is an annual discussion with the municipal management of primary health care regarding care for women experiencing violence. The fact that this action was not mentioned by the others may be related to the fact that they did not understand it as a comprehensive strategy for GNPs. Health service managers do not always have the appropriate technical training or a comprehensive understanding of the practices implemented by their teams. There is a need for technical support for managers so that they can assume the role and direct intervention and evaluation strategies based on the needs of the groups present in the territory^(24)^.

It is essential that managers understand that strategies for evaluating GNPs require knowledge of the field of Collective Health and that healthcare is provided within the geopolitical territory of social production and reproduction, since healthcare work aims to transform the population’s epidemiological profiles^(1)^.

The following were cited as indicators already in use: the number of reported cases of domestic violence and vulnerability indicators.

Highlighting the importance of reporting cases contradicts studies that discuss the underreporting of violence against women^(25,26)^. Notification is considered an important action for monitoring cases and can contribute to planning, implementing, and evaluating public policies to combat violence^(25)^.

The analysis of vulnerabilities was described by an interviewee, who made reference to the use of scales for this purpose. An indicator of vulnerability is not necessarily an indicator of GNP, however, identifying the vulnerability of women in a territory is fundamental to establishing actions to confront and overcome violence. Social inequalities, including gender power inequality, negatively impact affective and interpersonal relationships, access to rights, and the process of social reproduction^(27)^.

As cited by TIPESC, vulnerability indicators can support the evaluation of assistance that considers the living and working conditions of individuals and social groups, and can guide care actions in an equitable manner^(6)^. Furthermore, they enable the State to recognize weaknesses experienced by the population and contribute to the debate and implementation of policies to address them^(28)^.

The indicators listed in this research show strong convergence with the categories of GNP indicators described in an article that, although not dedicated to the study of GNP for women in situations of violence, investigates GNP indicators aimed at vulnerable groups^(6)^. The findings demonstrate a comprehensive and sensitive approach to social vulnerabilities. For instance, indicators such as “analysis of vulnerabilities: education, income, family composition, and dynamics” directly relate to the category “use of sociodemographic characteristics to estimate risks or vulnerabilities”. Meanwhile, the “analysis of women’s experience,” “adherence to care,” and “return rate to service(s)” reflect aspects of the care process that are strongly highlighted. This articulation reinforces the importance of nursing practices that are not only clinical, but also social, supportive, and interprofessional, especially in the care of women experiencing violence.

The analysis of the indicators listed by the participants regarding their relevance allowed their separation into three groups:

a) Assessment of women’s vulnerabilities based on sociodemographic characteristics, such as: income, education, family status, place of residence, employment, among others;
b) Assessment of trust building and engagement with the service: analysis of women’s experience with the service and return rate;
c) Evaluation of the care process: number of notifications, number of cases identified in PHC, number of referrals, adherence to guidelines, teamwork, resolution time, and case outcome.

Although the outcome was mentioned as an indicator, with the possibility of resulting in abandonment or discharge, no reference was found regarding its validation and application in the service. It is understood that the collection and analysis of this indicator would take place over the long term, as it is directly influenced by the follow-up period and is related to the woman’s adherence to the guidance and referrals made at the health unit and within the network.

As part of the assessment of the care process, indicators of both qualitative and quantitative nature were suggested. A quantitative approach is important for understanding the coverage, concentration, and efficiency of programs, actions, and interventions, and for evaluating specific objectives. Quantitative and qualitative approaches are not antagonistic, but complementary, so the combination of methods can contribute to understanding the reality that many communities experience when accessing a health service^(29)^.

Despite the complementarity of both approaches, the analysis of the indicators suggested and categorized as care process assessment does not suggest a defined methodological orientation. Thus, both the process and its evaluation can benefit from the construction of instruments with a defined theoretical and methodological orientation, such as TIPESC, to enable nurses to exercise a critical approach concerned with changing the current mode of social organization, policies for addressing violence, and practices for intervention in the phenomenon^(30)^.

Under the theoretical and methodological guidance of TIPESC, the development of an instrument is suggested to reinterpret the objective reality, previously captured and targeted by interventions, described in the category Good Nursing Practices for women in situations of domestic violence: bonding and support as protagonists of care in Primary Health Care. This way, the indicators suggested by the professionals can later form part of an instrument that allows for the evaluation of the product and the process.

This research was limited by its development during the COVID-19 pandemic, which required conducting interviews by telephone instead of in-person. Despite this, the willingness of the professionals to participate allowed for the collection of a large quantity and high quality of data.

It is believed that the results obtained favor the development of new research addressing the assessment of GNP from the perspective of Collective Health Nursing, as well as the development of assessment instruments focused on care practices for women in situations of violence. It should be noted that the results have the potential to be part of the intervention project development phase in objective reality, the third stage of TIPESC.

## CONCLUSION

Two indicators currently used to assess the GNPs to women experiencing violence have been identified: violence notification and vulnerability scale. The indicators mentioned are important, but insufficient when analyzed in isolation. The absence of a structured methodology guided by the principles of Collective Health limits the ability to explore and interpret important social issues that are part of the life and work context of the women served, which means that the evaluation of the GNP remains a challenge.

The study presents a scenario that, although permeated by care practices guided by a local protocol and structured public policies, still lacks the tools for conducting formal and targeted evaluations for service users, professionals, and managers.

Despite this, the proposal of indicators by the professionals themselves reveals potential for the collaborative construction of evaluative instruments that are more sensitive to the reality of the services and the users’ specific needs. Furthermore, TIPESC’s theoretical and methodological approach has demonstrated a great potential for linking practice and social critique, allowing for the reinterpretation of realities and the construction of effective interventions.

## Data Availability

The entire dataset supporting the results of this study is available upon request to the corresponding author.
