# Supplementary material for: Evaluation of Good Nursing Practices for women in situations of domestic violence
Source: Rev Esc Enferm USP. 2026 Jan 23;60:e20250374. doi: 10.1590/1980-220X-REEUSP-2025-0374en (PMC12854693; doi:10.1590/1980-220X-REEUSP-2025-0374en)
Supplement: Translation

# Translation: Avaliação das Boas Práticas de Enfermagem às mulheres em situação de violência doméstica*

**DOI:** 10.1590/1980-220X-REEUSP-2025-0374pt

**Authors:** Mariana Sbeghen Menegatti, Rosa Maria Godoy Serpa da Fonseca

**Affiliations:** 1 Universidade de São Paulo, Escola de Enfermagem, São Paulo, SP, Brasil.

**Keywords:** Enfermagem, Enfermagem Baseada em Evidências, Atenção Primária à Saúde, Indicadores de Qualidade em Assistência à Saúde, Violência Doméstica, Saúde Pública

## Abstract

**Objetivo:**

Identificar indicadores utilizados para avaliar as Boas Práticas de Enfermagem desenvolvidas na Atenção Primária à Saúde às mulheres em situação de violência doméstica.

**Método:**

Estudo qualitativo desenvolvido com enfermeiros da Atenção Primária de um município brasileiro. Foram desenvolvidas entrevistas semiestruturadas e para análise dos dados foi utilizado o *Software WebQDA*. As categorias analíticas utilizadas foram: violência de gênero e assistência de enfermagem segundo a Teoria de Intervenção Práxica de Enfermagem em Saúde Coletiva.

**Resultados:**

Emergiram as categorias empíricas: 1) Concepção da avaliação da assistência pelas usuárias do serviço; 2) Práticas avaliativas: ausência de estrutura avaliativa formal e a autoavaliação profissional; 3) Indicadores utilizados e potenciais para avaliação da assistência.

**Conclusão:**

Foram identificados dois indicadores já utilizados e houve a proposição de outros indicadores, destacando o potencial para a construção de instrumentos colaborativos. Apesar da existência de protocolos e políticas, faz-se necessária a instrumentação de uma avaliação, que pode ser beneficiada pela orientação teórico-metodológica da Teoria de Intervenção Práxica de Enfermagem Saúde Coletiva.

## INTRODUÇÃO

As Boas Práticas de Enfermagem (BPE) na Atenção Primária em Saúde (APS) possuem como característica o desenvolvimento de intervenções no território geopolítico de produção e reprodução social de indivíduos e coletividades. Os profissionais que as executam visam compreender e transformar os perfis de saúde-doença dos grupos sociais ali presentes. Ao prezar por uma atuação baseada na perspectiva da determinação social da saúde, têm como finalidade impactar as dimensões da realidade objetiva: singular (dos indivíduos), particular (dos grupos sociais) e estrutural (que corresponde às esferas mais gerais da sociedade)^(1)^.

Ao serem empregadas no enfrentamento da violência doméstica nos territórios, as BPE estruturam-se em ações orientadas pelo perfil epidemiológico dos grupos sociais presentes no território, incluindo o reconhecimento de seus modos de vida e de trabalho, além dos fenômenos que tornam as mulheres vulneráveis à violência. A captação e interpretação crítica da produção e reprodução social vivenciada pelas mulheres é imprescindível para o reconhecimento dos modos como a violência se determina, se manifesta e se reproduz^(2)^.

Os profissionais que atuam na APS reconhecem a importância da sua atuação e destacam a necessidade de diretrizes e definição clara de papeis, além de orientações baseadas em evidências, para o atendimento das mulheres em situação de violência, considerando o contexto em que se inserem. A estruturação de intervenções evidenciadas tem como fundamento a reflexão crítica constante, a partir de protocolos e diretrizes adequados à realidade local e do monitoramento de resultados a partir de avaliações^(3)^.

Na APS, avaliar é uma forma de orientação que contribui para a tomada de decisão e (re)direcionamento de ações que visam transformar a realidade de vida e saúde da população^(4)^. A avaliação dos serviços e das intervenções em saúde de modo geral vêm sendo alvo de pesquisas e destaca-se o reconhecimento de que estudos voltados à avaliação das intervenções na perspectiva da Saúde Coletiva são menos encontrados, e a avaliação de BPE voltadas às mulheres em situação de violência não é alvo específico de atenção^(5-7)^.

Considerando a lacuna de conhecimento relacionada aos instrumentos de avaliação de intervenções na perspectiva da Saúde Coletiva, compreende-se como importante o desenvolvimento de pesquisas que se voltem ao reconhecimento de indicadores ou instrumentos de avaliação da assistência à mulher em situação de violência doméstica.

Os indicadores são parâmetros, qualitativos ou quantitativos, que se destinam à análise de processos e resultados de uma determinada ação. Assim, são importantes instrumentos de gestão, que permitem monitorar situações que devem ser mudadas, incentivadas ou potencializadas^(5,8)^.

Identificar e analisar como enfermeiras(os) desenvolvem a avaliação da assistência que fornecem é importante para compreender o que orienta a prática profissional e os resultados dessa assistência. Portanto, o objetivo deste estudo foi identificar indicadores utilizados para avaliar as BPE desenvolvidas na APS às mulheres em situação de violência doméstica.

## MÉTODO

### Tipo de Estudo

Tratou-se de um estudo qualitativo que, visando garantir a qualidade e confiabilidade, foi realizado conforme preconizado pela ferramenta *Consolidated Criteria for Reporting Qualitative Research* (COREQ). Este artigo é produto de uma dissertação de mestrado defendida no Programa de Pós-graduação em Enfermagem da Escola de Enfermagem da Universidade de São Paulo^(9)^.

### Local e Período

A pesquisa foi realizada no Município de Araraquara, SP, Brasil, com coleta de dados entre 03 de junho e 15 de julho de 2022.

### População e Critérios de Seleção

A população alvo foram enfermeira(o)s atuantes na APS há pelo menos seis meses. A seleção dos participantes aconteceu por Amostragem em Bola de Neve. Foram inicialmente elencadas duas sementes, de modo aleatório, na lista de profissionais que faziam parte das equipes de APS do município e daí em diante a(o)s demais participantes foram sendo indicada(o)s pelas anteriores.

### Coleta de Dados

Para coleta de dados foi empregado um questionário constituído por perguntas de caracterização da(o)s participantes relacionadas à formação e ao trabalho. Após, foi realizada entrevista em profundidade seguindo roteiro semiestruturado que envolveu conhecimentos e experiências em relação às Boas Práticas de Enfermagem na APS às mulheres em situação de violência doméstica, além da indagação quanto às estratégias de avaliação empregadas.

Tanto o questionário de caracterização, quanto o roteiro semiestruturado foram testados em um piloto com uma profissional da APS do município que fazia parte da rede de contatos do grupo de pesquisa das autoras e que foi excluída da amostra. O teste permitiu ajuste do instrumento e adequação para o desenvolvimento da coleta de dados proposta.

Antes da realização dos convites para participação da pesquisa, e com o apoio da Secretaria de Saúde de Araraquara, foi realizado um momento de apresentação das pesquisadoras, do grupo de pesquisa, do projeto e dos objetivos do trabalho a ser desenvolvido. Na ocasião, foi aberto espaço para que a(o)s profissionais pudessem sanar dúvidas com as pesquisadoras e destacada a livre participação nas entrevistas, reiterando o anonimato daqueles que concordassem.

Posteriormente, a(o)s enfermeira(o)s foram contatadas(os) via telefone e convidadas(os) individualmente para participar. Destaca-se que as entrevistas semiestruturadas foram realizadas via telefone, dado o contexto da pandemia da COVID-19. Aquela(e) profissional que demonstrou interesse em participar, recebeu um e-mail contendo link de acesso ao Google Forms para preenchimento do Termo de Consentimento Livre e Esclarecido (TCLE) e escolheu data e horário para a entrevista, sendo orientada(o)s a escolher um ambiente privado, silencioso, livre de interrupções e no qual se sentissem confortáveis para realizar a entrevista. A duração das entrevistas foi de 56 minutos a 1hora e 42 minutos. Todos os profissionais contatados aceitaram e nenhum desistiu ao longo da coleta de dados.

As entrevistas foram conduzidas pela primeira autora, enfermeira, no período em questão pós-graduanda em curso de mestrado na área de Enfermagem em Saúde Coletiva. A supervisão da coleta de dados e demais etapas foi realizada pela segunda autora, enfermeira doutora, livre docente e orientadora no programa de pós-graduação citado. Ambas as autoras têm larga experiência em pesquisas qualitativas abrangendo, entre outros métodos de coleta de dados, as entrevistas semiestruturadas.

A coleta foi realizada até a saturação dos dados, alcançada com a 12ª entrevista. Após, foi realizado momento de discussão dos achados entre as autoras e foi acordada a realização de mais três entrevistas para confirmação da saturação, totalizando 15 profissionais entrevistada(o)s.

### Análise e Tratamento dos Dados

As informações foram armazenadas em dispositivo eletrônico acessado exclusivamente pelas pesquisadoras, sendo excluído qualquer registro armazenado na nuvem, garantindo o sigilo das informações. A(o)s participantes foram identificada(o)s como E1, E2, ...E15, garantindo o anonimato. Os dados de caracterização foram extraídos do Google Forms em planilha Excel e passaram por análise descritiva.

Os áudios das entrevistas foram transcritos, salvos em *Portable Document Format* (pdf) e submetidos a análise qualitativa, por meio da técnica de Análise de Conteúdo Temática, composta pelas etapas de: 1) pré-análise, 2) exploração do material 3) tratamento, interpretação dos resultados e inferência^(10)^. Contou-se com apoio do *Software Web Qualitative Data Analysis* (WEBQDA), disponibilizado em ambiente *online*, que por meio do sistema de codificação permitiu emergirem códigos que foram refinados e agrupados, dando origem à árvore de codificação^(11)^ da qual surgiram as categorias empíricas apresentadas nos resultados.

Os resultados foram discutidos utilizando-se as categorias analíticas: violência de gênero e assistência de enfermagem segundo a Teoria de Intervenção Práxica de Enfermagem em Saúde Coletiva (TIPESC)^(12)^.

A violência de gênero expressa-se em um conjunto de atos de violência praticados contra uma pessoa em razão de seu gênero, sendo que as mulheres são as vítimas hegemônicas dessa violência, e esses atos operam tanto de modo explícito como simbólico. A violência contra a mulher é uma das expressões da violência de gênero, dada a desigualdade imposta entre homens e mulheres e as normas socioculturais que conferem maior poder aos homens em relação às mulheres, inclusive nas relações que ocorrem nos espaços familiares ou em coabitações^(13)^.

A assistência de enfermagem segundo a TIPESC é orientada pela visão de mundo do materialismo histórico e dialético, que entende o processo saúde-doença como socialmente determinado. A TIPESC tem como bases filosóficas a historicidade e a dinamicidade e, como bases teóricas, as categorias conceituais (conjuntos totalizantes de noções e ideias historicamente construídas que demarcam em seus espaços as partes interligadas do fenômeno considerado) e categorias dimensionais (práxis, totalidade, interdependência entre o estrutural, o particular e o singular)^(12)^.

### Aspectos Éticos

Foi obedecida a Resolução 466, de 12 de dezembro de 2012, do Conselho Nacional de Saúde^(14)^ e as orientações para pesquisas em ambiente virtual, da Comissão Nacional de Ética em Pesquisa^(15)^. A coleta dos dados ocorreu mediante anuência no TCLE pela(o)s participantes. O projeto foi submetido ao Comitê de Ética em Pesquisa da Escola de Enfermagem da Universidade de São Paulo e autorizado em 25.02.2022, CAAE: 52878021.3.0000.5392, Parecer 5.266.104.

## RESULTADOS

Da(o)s 15 participantes, nove atuavam em Unidades de Saúde da Família e seis em outros serviços da APS, com tempo de atuação variando de 7 meses a 35 anos, sendo a média 13,9 anos. Todas(os) relataram o cuidado com mulheres em situação de violência ao longo de sua atuação na APS. Quatorze pessoas eram do sexo feminino e um do sexo masculino; onze eram casadas, duas divorciadas, uma em união estável e uma solteira; todas tinham filhos.

Quanto ao ano de término da graduação, o mais antigo foi 1977 e o mais recente 2020, com 60% entre 1991 e 2010. Catorze profissionais afirmaram ter concluído ou estar cursando especialização *lato sensu*. Quanto ao *stricto sensu*, três haviam concluído mestrado. Sete profissionais já tinham realizado algum curso sobre BPE na APS, com temas relacionados à imunização, humanização, violência contra a mulher, acolhimento, violência contra crianças e adolescentes, cuidados com populações vulneráveis, população em situação de rua, COVID-19, semiologia, amamentação, Política Nacional de Atenção Básica, atribuições do enfermeiro e cuidados baseados em protocolos.

Da análise qualitativa dos dados, emergiram as categorias empíricas: 1) A concepção da avaliação da assistência pelas usuárias do serviço; 2) Práticas avaliativas: a ausência de estrutura avaliativa formal e a autoavaliação profissional; 3) Indicadores utilizados e potenciais para avaliação da assistência.

### A Concepção da Avaliação da Assistência Pelas Usuárias do Serviço

A primeira categoria empírica revela obstáculos cognitivos e operacionais na implementação da avaliação da assistência recebida pelas usuárias. As(os) profissionais tinham dúvidas quanto à realização de alguma avaliação do atendimento pelas mulheres atendidas no serviço. Acreditavam que as mulheres não possuíam conhecimento sobre a avaliação da assistência recebida.

*Eu acho que elas não avaliam, eu acho que elas não têm esse conhecimento.* (E7)

Emergiu a possibilidade de avaliação do atendimento pela ouvidoria do município, porém não como sendo uma prática realizada por todas.

*Tem por meio da ouvidoria, mas não são todos que fazem.* (E15)

Uma resposta que os enfermeiros consideraram como uma avaliação da usuária, foi o comportamento da mulher mediante o atendimento, pelo vínculo formado e demonstração de confiança na equipe.

*Eu acho que é pelo feedback de vínculo, de vir procurar, de contar o que aconteceu. Assim, falar que elas agradecem, não, isso aí não, mas assim... vem, confia, conta o que aconteceu, o que fez, que está bem agora.* (E5)

*Práticas avaliativas: a ausência de estrutura avaliativa formal e a autoavaliação profissional*.

A segunda categoria inclui quais as formas de avaliação que estavam sendo usadas, mesmo que de forma não sistematizada.

Destacou-se a inexistência de uma avaliação formal pós-atendimento, bem como de instrumentos de avaliação.

*Não tem uma avaliação pós-atendimento, do que poderia ter feito melhor. Essa avaliação, de falar... Ah, esse atendimento, nós fizemos isso... Nós poderíamos ter melhorado? Eu nunca fiz no meu trabalho. Pelo menos nesse quesito de violência.* (E9)

*Não, eu não faço processo de avaliação. Eu faço atendimento, eu acompanho o atendimento, mas eu não tenho como avaliar.* (E8)

Apesar de negarem a existência de instrumentos formais de avaliação, a(o)s participantes destacaram a realização de autoavaliações, de forma empírica.

*Se há uma avaliação é mais uma autoavaliação(...) onde eu, muitas vezes, me questiono se estou fazendo aquilo que deveria fazer.* (E7)

*É empírico, faz por observação.* (E5)

A entrevistadora registrou que as perguntas relacionadas à avaliação geraram desconforto nos profissionais que precisavam de algum tempo para refletir sobre o que se compreendia como uma ação de avaliação para a sua prática profissional. Também foi registrado o receio em reconhecer que não eram empregadas estratégias de avaliação.

### Indicadores Utilizados e Potenciais Para Avaliação da Assistência

A última categoria mapeia instrumentos existentes e sugeridos. Uma entrevistada afirmou haver avaliação anual da assistência pela gestão da APS, quando enfermeira(o)s discutem o tema junto com a educação permanente do município e o cenário apresentado, bem como possíveis falhas.

*Eu sei que existe uma conversa que já vem de longa data. Esse protocolo vem se aprimorando e sei que existe sim uma avaliação periódica das ações. [...] Ali nos passam um cenário do município, quais são as formas de violência, onde a gente pode estar falhando, o que melhorar.* (E10)

A(o)s enfermeira(o)s citaram o número de notificações como indicador utilizado para mensurar os atendimentos, com possibilidade de aliá-lo ao monitoramento do que foi realizado.

*Olha, a gente tem uma notificação de violência [...] a gente alimenta o sistema e aí tem esse indicador de violência. A gente coloca todos os encaminhamentos, o que a gente fez, para poder dar a conduta adequada...* (E12)

Foi elencado como utilizado e essencial um indicador de vulnerabilidade social das mulheres envolvendo escolaridade, renda, constituição familiar e comorbidades.

*[...] um que seria essencial é o indicador de vulnerabilidade social. Algumas escalas de vulnerabilidade nós trabalhamos. Nós trabalhamos com o contexto familiar. Então, acredito que escolaridade, renda, constituição familiar, comorbidades foram coisas que nós vimos que, à medida que isso acomete mais a pessoa, principalmente eu, que atuo em uma região bem periférica, tem um impacto social que acaba refletindo nas ações em saúde.* (E6)

Uma participante destacou haver necessidade de um indicador que avalie a captação dos casos pela Atenção Básica, considerando o vínculo que o serviço tem com a comunidade.

*Então a AB, com o vínculo que ela tem com a comunidade, ela precisaria de um indicador que falasse da captação desses casos, e aí eu não consigo pensar ainda como a gente avaliaria isso.* (E10)

Foram sugeridos indicadores que permitissem avaliar a experiência da mulher na assistência, como acolhimento, dificuldades vivenciadas e o que foi relevante no atendimento.

*Eu acho que seria bem naquilo que ela sofreu. O que o atendimento nosso aqui ajudou [...] qual a pior dificuldade que ela teve.* (E4)

*Ah, eu acho que, talvez, uma conversa com ela, uma entrevista, através até de um instrumento que ela pudesse mostrar que ela conseguiu sair dessa situação ou que ela não conseguiu sair [...]* (E14)

O trabalho em equipe foi sugerido, apesar de ser considerado subjetivo e de difícil execução. Outra sugestão foi um indicador de encaminhamento para outros serviços da rede.

*Olha, indicador... Seria uma coisa mais subjetiva. Porque aí seria, por exemplo, uma avaliação de trabalho em equipe [...].* (E11)

*Talvez os encaminhamentos, para os serviços de referência, de proteção...* (E3)

Foram sugeridos também indicadores de adesão aos cuidados e desfecho, entre eles: superação ou não da violência, retomada da independência, vínculo empregatício, retorno à violência, abandono do acompanhamento ou alta.

*Eu acho que que os principais indicadores, para a gente conseguir, eu acho que seria a adesão mesmo...* (E12)

*Eu penso que é importante a gente saber a eficácia do atendimento. Então, se surtiu efeito, quantas você conseguiu retirar da violência, quantas voltaram para o estado de violência.* (E9)

*A taxa de abandono que eu tenho é uma coisa concreta [...]. Quantas fecharam o seguimento[...], e aí, quando eu fecho, eu fecho como alta....* (E8)

A possibilidade de sugerir indicadores que consideravam importantes e aplicáveis pareceu gerar conforto nos profissionais, de acordo com as observações da entrevistadora. Perceptivelmente, neste momento, a entrevista tornou-se mais fluida, possivelmente pelas sugestões serem uma ação de superação do desconforto que emergia do reconhecimento de não haver avaliação sistematizada.

Ainda, sobre os registros realizados pela pesquisadora, foi reconhecida uma diferença nas entrevistas das participantes mulheres em relação ao participante homem. A entrevista do enfermeiro teve duração de 56 minutos, em comparação à das enfermeiras que teve sempre mais de 1h e 16 minutos. Notou-se um maior envolvimento, inclusive emocional, e uma preocupação com o tema pelas mulheres entrevistadas ao discorrer sobre a assistência prestada.

A partir das respostas, foram compiladas 11 sugestões de indicadores, dois deles já utilizados e nove outros sugeridos, apresentados na Quadro 1.

**Quadro 1:** Indicadores utilizados e sugeridos para a avaliação das Boas Práticas de Enfermagem voltadas às mulheres em situação de violência na Atenção Primária à Saúde – São Paulo, SP, Brasil, 2025.

Indicadores
Número de notificações de casos de violência doméstica
Número de casos de violência identificados na Atenção Primária à Saúde
Número de encaminhamentos realizados
Número de mulheres atendidas nos serviços da rede encaminhadas pela Atenção Primária à Saúde
Adesão aos cuidados, orientações
Análise da experiência da mulher (acolhimento ou dificuldades enfrentadas)
Trabalho em equipe (profissionais que atenderam e intervieram sobre a situação vivenciada pela mulher)
Análise das vulnerabilidades: escolaridade, renda, composição e dinâmica familiar
Taxa de retorno ao(s) serviço(s)
Tempo para resolução da situação que está em atendimento
Desfecho do caso: abandono ou alta

## DISCUSSÃO

A caracterização das(os) participantes evidencia que estas(es) possuem, em maioria, ampla experiência na APS, o que pode favorecer a identificação e o manejo de situações complexas, como a violência contra a mulher. O predomínio do sexo feminino reflete a composição majoritariamente feminina da enfermagem, frequentemente associada às práticas de cuidado e acolhimento. A diversidade quanto ao tempo de formação e de atuação sugere a coexistência de diferentes gerações profissionais, o que pode ampliar as perspectivas sobre o tema. O número de profissionais com especialização *lato sensu* e a presença de mestres indica investimento na formação continuada, enquanto a participação em cursos sobre BPE, incluindo temas relacionados à violência contra a mulher, demonstra busca por qualificação para o cuidado às mulheres.

A(o)s profissionais entrevistadas fizeram suposições quanto à realização de uma avaliação pelas mulheres atendidas, inclusive sugerindo que não realizavam e que elas não possuem conhecimento para isso.

O conhecimento das mulheres envolve, para além do seu nível de formação, a compreensão do direito à saúde, da qualidade dos serviços, e dos mecanismos de responsabilização e eficácia, que pode ser influenciado pelo grupo social em que se inserem estas mulheres e a comunidade em que se desenvolvem^(16)^. Ou seja, tanto o impacto das ações desenvolvidas quanto a crítica sobre elas têm influência direta do contexto social em que são realizadas^(17)^.

Por exemplo, a avaliação de um serviço pode encontrar interferências quando as mulheres atendidas não possuem conhecimento sobre os seus direitos dentro do sistema de saúde ou questões como acolhimento ou privacidade dentro do serviço. Deste modo, a mulher pode não saber que existe a possibilidade de manifestar elogios, críticas ou sugestões, ou pode compreender que o que lhe foi oferecido foi o possível, e este possível, na realidade, pode ter sido aquém do preconizado em políticas ou protocolos.

Ferramentas ou estratégias que envolvem a comunidade com o objetivo de fortalecer a relação desta com os profissionais, são facilitadores e promovem a criação de oportunidades para identificar obstáculos ou soluções para melhorar a assistência oferecida dentro dos serviços de saúde, o que é parte fundamental para a garantia da responsabilidade social do usuário^(16)^.

Entre os achados, algumas profissionais destacaram a viabilidade de a mulher realizar a avaliação da assistência que recebe por meio de canais institucionais, como a ouvidoria do município. Trata-se de um dispositivo constitucional democratizante que permite a escuta direta da população e possui como funções dar voz ativa aos indivíduos, permitindo críticas, sugestões ou denúncias, possibilitar a execução do controle social por meio da fiscalização e avaliar a satisfação do usuário^(18)^. Cabe questionar a sua divulgação e instigar os serviços a propagarem a existência da ouvidoria e seu objetivo, bem como o acesso ao canal por diferentes estratégias, evitando meios que limitem o alcance de determinados grupos sociais, como o acesso exclusivo por páginas online, redes sociais ou telefone.

A(o)s enfermeira(o)s descreveram que uma forma pela qual reconhecem a avaliação da mulher sobre a assistência prestada era a aproximação da mulher com a equipe e a expressão da gratidão. Estudo que se dedica a analisar indicadores da assistência afirma que a adesão ao plano terapêutico-cuidativo e o aumento da confiança nos profissionais podem ser considerados para avaliação das BPE^(5)^.

Em consonância com a dúvida sobre a realização da avaliação pelas mulheres e a experiência destas nos serviços, foi sugerido pelas profissionais e emerge na terceira categoria empírica a avaliação da experiência das mulheres ao passarem por atendimento. A experiência antes, durante e depois do atendimento, além de como e quando a pessoa utiliza e necessita do serviço de saúde, são aspectos que incidem diretamente sobre a satisfação. Apesar da influência que a experiência tem sobre a satisfação, é importante destacar que satisfação é um conceito relativo, influenciado por aspectos como a expectativa e a percepção da pessoa quanto aos serviços recebidos e em relação aos profissionais que a atenderam^(19)^. A pessoa pode estar satisfeita mesmo sem ter tido uma boa experiência, o que pode levá-la a não voltar mais ao serviço^(20)^.

A experiência do usuário recebeu atenção do Ministério da Saúde brasileiro em Portaria que dispõe sobre indicadores para pagamento por desempenho da APS, para os anos de 2021 e 2022, incluindo indicadores de avaliação da qualidade assistencial e experiência do paciente^(21)^. O manual de Autoavaliação para Melhoria do Acesso e da Qualidade (AMAQ), de caráter reflexivo e problematizador, inclui no julgamento do processo de fazer saúde, além de profissionais e gestores, o usuário do serviço no que tange a participação, o controle social e a satisfação^(22)^. Apesar disso, na presente pesquisa, não foram citadas estratégias ou itens de avaliação propostos nos documentos citados.

Quanto à realização de avaliações gerenciais das BPE, emergiram dúvidas quanto à uma execução formalizada. Corrobora, assim, o que a literatura evidencia como lacuna na implementação de avaliações sobre as práticas dos profissionais de saúde. A avaliação ainda constitui um desafio para a maioria dos profissionais que atuam, dada a complexidade do objeto^(5)^.

Destacou-se a realização de autoavaliações, de forma empírica, pelos profissionais. Visto que a autoavaliação citada não cumpre preceitos direcionadores, torna-se um processo reflexivo do profissional sobre sua atuação. É importante que a ação de autoavaliação seja formalizada e direcionada, para que a(o)s profissionais se percebam dentro de um processo seguro de mudança que implica movimento, escuta e consideração de possibilidades que contribuirá para o planejamento de ações futuras, apontando novos raciocínios e novas soluções^(23)^.

Uma enfermeira afirmou haver uma discussão anual sobre a atenção às mulheres em situação de violência com a gestão municipal da APS. O fato dessa ação não ter sido citada pelos demais pode ser relacionada ao fato de não a compreenderem como uma estratégia abrangente às BPEs. Nem sempre os gestores de serviços de saúde possuem formação técnica apropriada ou compreensão abrangente sobre as práticas implementadas pelas equipes. Há necessidade de apoio técnico aos gestores para que assumam a função e direcionem estratégias de intervenção e avaliação sobre as necessidades dos grupos presentes no território^(24)^.

É fundamental que os gestores compreendam que estratégias de avaliação de BPEs exigem conhecimentos sobre o campo da Saúde Coletiva e que a assistência se realiza no território geopolítico de produção e reprodução social, uma vez que o trabalho em saúde visa à transformação dos perfis epidemiológicos da população^(1)^.

Foram citados como indicadores já utilizados: o número de notificações de casos de violência doméstica e indicadores de vulnerabilidade.

A sinalização da importância da notificação dos casos contradiz estudos que discutem a subnotificação da violência contra a mulher^(25,26)^. A notificação é considerada uma importante ação para monitoramento dos casos e pode contribuir para planejar, implementar e avaliar as políticas públicas de enfrentamento à violência^(25)^.

A análise das vulnerabilidades foi descrita por uma entrevistada, que fez referência ao uso de escalas com este objetivo. Um indicador de vulnerabilidade não é necessariamente um indicador de BPE, entretanto, identificar a vulnerabilidade das mulheres de um território é fundamental para estabelecer ações de enfrentamento e superação da violência. As desigualdades sociais, inclusive a desigualdade de poder entre os gêneros, impacta negativamente as relações afetivas e interpessoais, no acesso aos direitos e no processo de reprodução social^(27)^.

Conforme citado pela TIPESC, os indicadores de vulnerabilidade podem apoiar a avaliação da assistência que considera as condições de vida e trabalho dos indivíduos e dos grupos sociais, podendo guiar as ações de cuidado de maneira equitativa^(6)^. Além disso, possibilitam ao Estado reconhecer fragilidades vivenciadas pela população e contribuir para o debate e implementação de políticas de enfrentamento^(28)^.

Os indicadores elencados nesta pesquisa apresentam forte convergência com as categorias de indicadores de BPE descritas em artigo que, embora não se dedique ao estudo das BPE às mulheres em situação de violência, investiga indicadores de BPE voltadas a grupos vulneráveis^(6)^. Os achados evidenciam uma abordagem integral e sensível às vulnerabilidades sociais. Por exemplo, indicadores como “análise das vulnerabilidades: escolaridade, renda, composição e dinâmica familiar” dialogam diretamente com a categoria “uso de características sociodemográficas para estimar riscos ou vulnerabilidades”. Já a “análise da experiência da mulher”, “adesão aos cuidados” e “taxa de retorno ao(s) serviço(s)” refletem aspectos do processo assistencial fortemente destacado. Essa articulação reforça a importância de práticas de enfermagem que sejam não apenas clínicas, mas também sociais, acolhedoras e interprofissionais, especialmente no cuidado a mulheres em situação de violência.

A análise dos indicadores listados pelas participantes quanto à pertinência possibilitou separá-los em três grupos:

a) Avaliação de vulnerabilidades das mulheres a partir de características sociodemográficas, como: renda, escolaridade, condição familiar, local de residência, emprego, entre outros;
b) Avaliação da construção da confiança e do vínculo com o serviço: análise da experiência da mulher no serviço e taxa de retorno;
c) Avaliação do processo de cuidado: número de notificações, número de casos identificados na APS, número de encaminhamentos, adesão às orientações, trabalho em equipe, tempo de resolução e desfecho do caso.

Apesar da menção do desfecho como um indicador, com a possibilidade de resultar em abandono ou alta, não foi encontrada referência quanto à sua validação e aplicação no serviço. Compreende-se que a coleta e análise desse indicador aconteceria em longo prazo, pois é diretamente influenciado pelo período de acompanhamento e está relacionado com a adesão da mulher às orientações e aos encaminhamentos realizados na unidade de saúde e na rede.

Como avaliação do processo de cuidado, houve sugestão de indicadores de cunho qualitativo e quantitativo. A abordagem quantitativa é importante para conhecer cobertura, concentração e eficiência de programas, ações, intervenções e para avaliar objetivos específicos. As aproximações quantitativas e qualitativas não são antagônicas, mas complementares, de modo que a combinação de métodos pode contribuir para reconhecer a realidade que muitas comunidades vivem ao acessar um serviço de saúde^(29)^.

Apesar da complementaridade de ambas as vertentes, a análise dos indicadores sugeridos e categorizados como avaliação de processo de cuidado não sugere uma orientação metodológica definida. Deste modo, tanto o processo quanto a sua avaliação podem ser beneficiados pela construção de instrumentos com orientação teórico-metodológica definida, a exemplo da TIPESC, para possibilitar à(o) enfermeira(o) o exercício de uma crítica preocupada com a mudança do atual modo de organização da sociedade, das políticas de enfrentamento da violência e das práticas para a intervenção no fenômeno^(30)^.

Sob a orientação teórico-metodológica da TIPESC, convém a composição de um instrumento com o objetivo de reinterpretar a realidade objetiva, antes captada e alvo de intervenções, descritas na categoria Boas Práticas de Enfermagem às mulheres em situação de violência doméstica: vínculo e acolhimento como protagonistas da assistência na Atenção Primária à Saúde. Desta forma, os indicadores sugeridos pela(o)s profissionais podem futuramente compor um instrumento que permita a avaliação do produto e do processo.

Esta pesquisa apresentou como limitações o seu desenvolvimento durante a pandemia da COVID-19, o que exigiu a realização das entrevistas via telefone, em substituição à entrevista presencial. Apesar disso, a disposição dos profissionais para participar permitiu que fossem coletados dados em grande quantidade e qualidade.

Acredita-se que os resultados obtidos favorecem o desenvolvimento de novas pesquisas que abordam a avaliação de BPE sob a perspectiva da Enfermagem em Saúde Coletiva, bem como o desenvolvimento de instrumentos de avaliação voltados às práticas assistenciais a mulheres em situação de violência. Destaca-se que os resultados têm potencial para compor a etapa de construção de projeto de intervenção na realidade objetiva, a terceira etapa da TIPESC.

## CONCLUSÃO

Foram identificados dois indicadores atualmente utilizados para avaliação das BPE às mulheres em situação de violência, quais sejam, notificação da violência e escala de vulnerabilidade. Os indicadores mencionados são importantes, mas insuficientes quando analisados isoladamente. A ausência de uma metodologia estruturada e orientada pelos preceitos da Saúde Coletiva limita a capacidade de explorar e interpretar questões sociais importantes que se inserem no contexto de vida e trabalho das mulheres atendidas, o que faz com que a avaliação das BPE permaneça como um desafio.

O estudo apresenta um cenário que, embora permeado por práticas assistenciais orientadas por um protocolo local e políticas públicas estruturadas, ainda carece de instrumentalização para a realização de avaliações formais e direcionadas, para as usuárias dos serviços, para os profissionais e para os gestores.

Apesar disso, a proposição de indicadores pelos próprios profissionais revela potencial para a construção colaborativa de instrumentos avaliativos mais sensíveis à realidade dos serviços e às especificidades das usuárias. Além disso, a orientação teórico-metodológica da TIPESC demonstrou grande possibilidade de articulação entre a prática e a crítica social, permitindo reinterpretar realidades e construir intervenções eficazes.

## Data Availability

Todo o conjunto de dados que dá suporte aos resultados deste estudo foi publicado no próprio artigo.

# Translation: Evaluación de las Buenas Prácticas de Enfermería para mujeres en situaciones de violencia doméstica*

**DOI:** 10.1590/1980-220X-REEUSP-2025-0374es

**Authors:** Mariana Sbeghen Menegatti, Rosa Maria Godoy Serpa da Fonseca

**Affiliations:** 1 Universidade de São Paulo, Escola de Enfermagem, São Paulo, SP, Brasil.

**Keywords:** Enfermería, Enfermería Basada en la Evidencia, Atención Primaria de Salud, Indicadores de Calidad de la Atención de Salud, Violencia Doméstica, Salud Pública

## Abstract

**Objetivo:**

Identificar los indicadores utilizados para evaluar las Buenas Prácticas de Enfermería desarrolladas en la Atención Primaria de Salud para mujeres en situaciones de violencia doméstica.

**Método:**

Estudio cualitativo realizado con enfermeros de atención primaria en un municipio brasileño. Se elaboraron entrevistas semiestructuradas y se utilizó el Software WebQDA para el análisis de datos. Las categorías analíticas utilizadas fueron: violencia de género y cuidados de enfermería según la Teoría de la Intervención en la Praxis de Enfermería en Salud Colectiva.

**Resultados:**

Surgieron categorías empíricas: 1) Concepción de la evaluación del servicio por parte de los usuarios del servicio; 2) Prácticas de evaluación: ausencia de una estructura de evaluación formal y autoevaluación profesional; 3) Indicadores utilizados y potenciales para evaluar la atención.

**Conclusión:**

Se identificaron dos indicadores que ya están en uso y se propusieron otros, lo que pone de relieve el potencial para construir instrumentos colaborativos. A pesar de la existencia de protocolos y políticas, es necesaria una herramienta de evaluación, que puede beneficiarse de la orientación teórica y metodológica de la Teoría de la Intervención en la Práctica de Enfermería en Salud Colectiva.

## INTRODUCCIÓN

Las buenas prácticas de enfermería (BPE) en la atención primaria en salud (APS) se caracterizan por el desarrollo de intervenciones en el territorio geopolítico de producción y reproducción social de individuos y colectividades. Los profesionales que las ejecutan buscan comprender y transformar los perfiles de salud-enfermedad de los grupos sociales presentes en ese territorio. Al valorar una actuación basada en la perspectiva de la determinación social de la salud, tienen como objetivo impactar las dimensiones de la realidad objetiva: singular (de los individuos), particular (de los grupos sociales) y estructural (que corresponde a las esferas más generales de la sociedad)^(1)^.

Al emplearse para hacer frente a la violencia doméstica en los territorios, las BPE se estructuran en acciones orientadas por el perfil epidemiológico de los grupos sociales presentes en el territorio, incluyendo el reconocimiento de sus modos de vida y de trabajo, además de los fenómenos que hacen a las mujeres vulnerables a la violencia. La captación y la interpretación crítica de la producción y reproducción social que experimentan las mujeres es imprescindible para reconocer las formas en que se determina, se manifiesta y se reproduce la violencia^(2)^.

Los profesionales que trabajan en la APS reconocen la importancia de su actuación y destacan la necesidad de directrices y una definición clara de funciones, además de orientaciones basadas en la evidencia, para la atención a las mujeres en situación de violencia, considerando el contexto en el que se insertan. La estructuración de las intervenciones evidenciadas se basa en la reflexión crítica constante, a partir de protocolos y directrices adecuados a la realidad local y del seguimiento de los resultados a partir de evaluaciones^(3)^.

En la APS, la evaluación es una forma de orientación que contribuye a la toma de decisiones y a la (re)orientación de las acciones que visan transformar la realidad de la vida y la salud de la población^(4)^. La evaluación de los servicios y las intervenciones en salud en general ha sido objeto de investigaciones y se destaca el reconocimiento de que los estudios orientados a la evaluación de las intervenciones desde la perspectiva de la salud colectiva son menos frecuentes, y la evaluación de las BPE dirigidas a las mujeres en situación de violencia no es un objetivo específico de atención^(5-7)^.

Teniendo en cuenta las brechas de conocimiento relacionadas con los instrumentos de evaluación de las intervenciones desde la perspectiva de la salud colectiva, se considera importante el desarrollo de investigaciones que se centren en el reconocimiento de indicadores o instrumentos de evaluación de la asistencia a las mujeres en situación de violencia doméstica.

Los indicadores son parámetros, cualitativos o cuantitativos, destinados al análisis de los procesos y resultados de una determinada acción. Por lo tanto, son instrumentos de gestión importantes, que permiten monitorear situaciones que deben cambiarse, incentivarse o potenciarse^(5,8)^.

Identificar y analizar cómo las enfermeras y enfermeros evalúan la asistencia que prestan es importante para comprender qué orienta la práctica profesional y los resultados de dicha asistencia. Por lo tanto, el objetivo de este estudio fue identificar los indicadores utilizados para evaluar las BPE desarrolladas en la APS a las mujeres en situación de violencia doméstica.

## MÉTODO

### Tipo de Estudio

Se trata de un estudio cualitativo que, con el fin de garantizar la calidad y la confiabilidad, se llevó a cabo de acuerdo con lo recomendado por la herramienta*Criterios consolidados para la presentación de informes de investigación cualitativa* (COREQ). Este artículo es el resultado de una tesis de maestría defendida en el Programa de Posgrado en Enfermería de la Escuela de Enfermería de la Universidad de São Paulo^(9)^.

### Lugar y Período

La investigación se llevó a cabo en el municipio de Araraquara, SP, Brasil, y los datos se recopilaron entre el 3 de junio y el 15 de julio de 2022.

### Población y Criterios de Selección

La población objetivo eran enfermeros que llevaban al menos seis meses trabajando en la APS. La selección de los participantes se realizó mediante muestreo en bola de nieve. Inicialmente se seleccionaron dos semillas, de forma aleatoria, en la lista de profesionales que formaban parte de los equipos de APS del municipio y, a partir de ahí, los demás participantes fueron indicados por los anteriores.

### Recolección de Datos

Para la recolección de datos se utilizó un cuestionario compuesto por preguntas de caracterización de los participantes relacionadas con la formación y el trabajo. A continuación, se realizó una entrevista en profundidad siguiendo un guion semiestructurado que incluía conocimientos y experiencias en relación con las buenas prácticas de enfermería en la APS para mujeres en situación de violencia doméstica, además de preguntas sobre las estrategias de evaluación empleadas.

Tanto el cuestionario de caracterización como el guion semiestructurado se probaron en una prueba piloto con una profesional de la APS del municipio que formaba parte de la red de contactos del grupo de investigación de las autoras y que fue excluida de la muestra. La prueba permitió ajustar el instrumento y adaptarlo para el desarrollo de la recopilación de datos propuesta.

Antes de realizar las invitaciones para participar en la investigación, y con el apoyo de la Secretaría de Salud de Araraquara, se llevó a cabo una presentación de las investigadoras, el grupo de investigación, el proyecto y los objetivos del trabajo a desarrollar. En esa ocasión, se abrió un espacio para que los profesionales pudieran aclarar sus dudas con las investigadoras y se destacó la libre participación en las entrevistas, reiterando el anonimato de aquellos que aceptaran participar.

Posteriormente, se contactó a las enfermeras por teléfono y se las invitó individualmente a participar. Cabe destacar que las entrevistas semiestructuradas se realizaron por teléfono, dado el contexto de la pandemia de COVID-19. Los profesionales que mostraron interés en participar recibieron un correo electrónico con un enlace de acceso a Google Forms para completar el Término de Consentimiento Libre e Informado (TCLI) y eligieron la fecha y la hora de la entrevista, con la recomendación de elegir un entorno privado, silencioso, libre de interrupciones y en el que se sintieran cómodos para realizar la entrevista.

La duración de las entrevistas fue de 56 minutos a 1 hora y 42 minutos. Todos los profesionales contactados aceptaron y ninguno se retiró durante la Recolección de datos. Las entrevistas fueron realizadas por la primera autora, enfermera, en el período en cuestión, estudiante de posgrado en un máster en el área de Enfermería en Salud Colectiva.

La supervisión de la recopilación de datos y otras etapas fue realizada por la segunda autora, enfermera con doctorado, profesora y tutora en el programa de posgrado mencionado. Ambas autoras tienen una amplia experiencia en investigaciones cualitativas que abarcan, entre otros métodos de recopilación de datos, las entrevistas semiestructuradas.

La recopilación se llevó a cabo hasta la saturación de los datos, alcanzada con la duodécima entrevista. A continuación, se llevó a cabo un debate sobre los resultados entre las autoras y se acordó realizar tres entrevistas más para confirmar la saturación, con un total de 15 profesionales entrevistados.

### Análisis y Tratamiento de Los Datos

La información se almacenó en un dispositivo electrónico al que solo tenían acceso las investigadoras, excluyéndose cualquier registro almacenado en la nube, lo que garantizó la confidencialidad de la información. Los participantes fueron identificados como E1, E2, ...E15, garantizando el anonimato. Los datos de caracterización se extrajeron de Google Forms en una hoja de cálculo de Excel y se sometieron a un análisis descriptivo.

Las grabaciones de audio de las entrevistas se transcribieron, se guardaron en formato PDF (Portable Document Format) y se sometieron a un análisis cualitativo mediante la técnica de análisis de contenido temático, que consta de las siguientes etapas: 1) preanálisis, 2) exploración del material, 3) tratamiento, interpretación de los resultados e inferencia^(10)^. Se contó con el apoyo del Software Web Qualitative Data Analysis (WEBQDA), disponible en un entorno en línea, que mediante el sistema de codificación permitió que surgieran códigos que fueron refinados y agrupados, dando lugar al árbol de codificación^(11)^del que surgieron las categorías empíricas presentadas en los resultados.

Los resultados se discutieron utilizando las categorías analíticas: violencia de género y asistencia de enfermería según la Teoría de la Intervención Práctica de Enfermería en Salud Colectiva (TIPESC)^(12)^.

La violencia de género se expresa en un conjunto de actos de violencia practicados contra una persona por razón de su género, siendo las mujeres las víctimas hegemónicas de esta violencia, y estos actos operan tanto de manera explícita como simbólica. La violencia contra la mujer es una de las expresiones de la violencia de género, dada la desigualdad impuesta entre hombres y mujeres y las normas socioculturales que otorgan mayor poder a los hombres en relación con las mujeres, incluso en las relaciones que se dan en los espacios familiares o en las convivencias^(13)^.

La asistencia de enfermería según la TIPESC se orienta por la visión del mundo del materialismo histórico y dialéctico, que entiende el proceso salud-enfermedad como socialmente determinado. La TIPESC tiene como bases filosóficas la historicidad y la dinámica y, como bases teóricas, las categorías conceptuales (conjuntos totalizantes de nociones e ideas construidas históricamente que demarcan en sus espacios las partes interconectadas del fenómeno considerado) y las categorías dimensionales (praxis, totalidad, interdependencia entre lo estructural, lo particular y lo singular)^(12)^.

### Aspectos Éticos

Se cumplió con la Resolución 466, de 12 de diciembre de 2012, del Consejo Nacional de Salud^(14)^y las orientaciones para investigaciones en ambiente virtual, de la Comisión Nacional de Ética en Investigación^(15)^. La recolección de datos se realizó mediante el consentimiento informado de los participantes. El proyecto fue sometido al Comité de Ética en Investigación de la Escuela de Enfermería de la Universidad de São Paulo y autorizado el 25 de febrero de 2022, CAAE: 52878021.3.0000.5392, Dictamen 5.266.104.

## RESULTADOS

De los 15 participantes, nueve trabajaban en Unidades de Salud Familiar y seis en otros servicios de APS, con una antigüedad que oscilaba entre 7 meses y 35 años, con una media de 13,9 años.

Todos informaron haber atendido a mujeres en situación de violencia a lo largo de su actuación en la APS. Catorce personas eran mujeres y una era hombre; once estaban casadas, dos divorciadas, una en unión libre y una soltera; todas tenían hijos.

En cuanto al año de finalización de la licenciatura, el más antiguo fue 1977 y el más reciente 2020, con un 60 % entre 1991 y 2010. Catorce profesionales afirmaron haber completado o estar cursando una especialización*lato sensu*. En cuanto al*stricto sensu*, tres habían completado una maestría. Siete profesionales ya habían realizado algún curso sobre BPE en APS, con temas relacionados con la inmunización, la humanización, la violencia contra la mujer, la acogida, la violencia contra los niños y adolescentes, la atención a poblaciones vulnerables, la población viviendo en la calle, la COVID-19, la semiología, la lactancia materna, la Política Nacional de Atención Básica, las atribuciones del enfermero y la atención basada en protocolos.

Del análisis cualitativo de los datos surgieron las siguientes categorías empíricas: 1) La concepción de la evaluación de la asistencia por parte de las usuarias del servicio; 2) Prácticas evaluativas: la ausencia de una estructura evaluativa formal y la autoevaluación profesional; 3) Indicadores utilizados y potenciales para la evaluación de la asistencia.

### La Concepción de la Evaluación de la Asistencia por Parte de las Usuarias del Servicio

La primera categoría empírica revela obstáculos cognitivos y operativos en la implementación de la evaluación de la asistencia recibida por las usuarias. Los profesionales tenían dudas sobre la realización de alguna evaluación de la atención por parte de las mujeres atendidas en el servicio. Creían que las mujeres no tenían conocimientos sobre la evaluación de la asistencia recibida.

*Creo que no evalúan, creo que no tienen ese conocimiento.*(E7;)

Surgió la posibilidad de evaluar la atención por parte de la oficina del ombudsman del municipio, pero no como una práctica realizada por todas.

*Se hace a través de la oficina del ombudsman, pero no todas lo hacen.*(E1;5;)

Una respuesta que los enfermeros consideraron como una evaluación de la usuaria fue el comportamiento de la mujer durante la atención, por el vínculo formado y la demostración de confianza en el equipo.

*Creo que es por el feedback del vínculo, por venir a buscar, por contar lo que pasó. Así, decir que agradecen, no, eso no, pero así... viene, confía, cuenta lo que pasó, lo que hizo, que ahora está bien.*(E5;)

*Prácticas de evaluación: la ausencia de una estructura de evaluación formal y la autoevaluación professional.*

La segunda categoría incluye las formas de evaluación que se estaban utilizando, aunque de manera no sistemática.

Se destacó la inexistencia de una evaluación formal posterior a la atención, así como de instrumentos de evaluación.

*No hay una evaluación posterior a la atención, de lo que se podría haber hecho mejor. Esa evaluación, de hablar... Ah, esa atención, hicimos eso... ¿Podríamos haber mejorado? Nunca lo he hecho en mi trabajo. Al menos en lo que respecta a la violencia.*(E9;)

*No, no hago un proceso de evaluación. Presto atención, acompaño la atención, pero no tengo cómo evaluar.*(E8;)

A pesar de negar la existencia de instrumentos formales de evaluación, los participantes destacaron la realización de autoevaluaciones, de forma empírica.

*Si hay una evaluación, es más bien una autoevaluación (...) en la que, a menudo, me pregunto si estoy haciendo lo que debería hacer.*(E7;)

*Es empírico, se hace por observación.*(E5;)

La entrevistadora registró que las preguntas relacionadas con la evaluación generaron incomodidad en los profesionales, que necesitaban algo de tiempo para reflexionar sobre lo que se entendía como una acción de evaluación para su práctica profesional. También se registró el temor a reconocer que no se empleaban estrategias de evaluación.

### Indicadores Utilizados y Potenciales Para la Evaluación de la Atención

La última categoría mapea los instrumentos existentes y sugeridos. Una entrevistada afirmó que existe una evaluación anual de la asistencia por parte de la gestión de la APS, cuando las enfermeras discuten el tema junto con la educación permanente del municipio y el escenario presentado, así como las posibles fallas.

*Sé que existe una conversación que viene desde hace mucho tiempo. Este protocolo se ha ido perfeccionando y sé que sí existe una evaluación periódica de las acciones. [...] Allí nos presentan un panorama del municipio, cuáles son las formas de violencia, dónde podemos estar fallando, qué mejorar.*(E1;0;)

Las enfermeras mencionaron el número de notificaciones como indicador utilizado para medir la atención, con la posibilidad de añadirlo al seguimiento de lo que se ha realizado.

*Mira, tenemos una notificación de violencia [...] alimentamos el sistema y ahí está ese indicador de violencia. Ponemos todas las derivaciones, lo que hicimos, para poder dar la conducta adecuada...*(E12)

Se enumeró como utilizado y esencial un indicador de vulnerabilidad social de las mujeres que involucra la escolaridad, los ingresos, la constitución familiar y las comorbilidades.

*[...] uno que sería esencial es el indicador de vulnerabilidad social. Trabajamos con algunas escalas de vulnerabilidad. Trabajamos con el contexto familiar. Entonces, creo que la escolaridad, los ingresos, la constitución familiar y las comorbilidades fueron cosas que vimos que, a medida que esto afecta más a la persona, principalmente a mí, que trabajo en una región muy periférica, tiene un impacto social que termina reflejándose en las acciones de salud.*(E6;)

Una participante destacó la necesidad de un indicador que evalúe la captación de casos por parte de la Atención Primaria, considerando el vínculo que el servicio tiene con la comunidad.

*Entonces, la AP, con el vínculo que tiene con la comunidad, necesitaría un indicador que hablara de la captación de estos casos, y ahí todavía no consigo pensar cómo evaluaríamos eso.*(E1;0;)

Se sugirieron indicadores que permitieran evaluar la experiencia de la mujer en la atención, como la acogida, las dificultades experimentadas y lo que fue relevante en la atención.

*Creo que sería en lo que ella sufrió. En qué la ayudó nuestra atención aquí [...] cuál fue la peor dificultad que tuvo.*(E4;)

*Ah, creo que, tal vez, una conversación con ella, una entrevista, incluso a través de un instrumento que le permitiera demostrar que logró salir de esa situación o que no logró salir [...]* (E14)

Se sugirió el trabajo en equipo, a pesar de que se considera subjetivo y difícil de ejecutar. Otra sugerencia fue un indicador de derivación a otros servicios de la red.

*Mira, indicador... Sería algo más subjetivo. Porque ahí sería, por ejemplo, una evaluación del trabajo en equipo [...].*(E1;1;)

*Quizás las derivaciones a los servicios de referencia, de protección...*(E3)

También se sugirieron indicadores de adherencia a la atención y resultados, entre ellos: superación o no de la violencia, recuperación de la independencia, vínculo laboral, retorno a la violencia, abandono del seguimiento o alta.

*Creo que los principales indicadores, para que podamos lograrlo, creo que sería la adherencia...*(E12)

*Creo que es importante que sepamos la eficacia de la atención. Entonces, si surtió efecto, cuántas lograste sacar de la violencia, cuántas volvieron al estado de violencia.*(E9;)

*La tasa de abandono que tengo es algo concreto [...]. Cuántas cerraron el seguimiento [...], y entonces, cuando cierro, cierro como alta...*(E8)

La posibilidad de sugerir indicadores que consideraban importantes y aplicables pareció generar comodidad en los profesionales, según las observaciones de la entrevistadora. Perceptiblemente, en ese momento, la entrevista se volvió más fluida, posiblemente porque las sugerencias eran una acción para superar la incomodidad que surgía del reconocimiento de que no había una evaluación sistematizada.

Además, en los registros realizados por la investigadora, se reconoció una diferencia en las entrevistas de las participantes mujeres en relación con el participante hombre. La entrevista al enfermero duró 56 minutos, en comparación con las de las enfermeras, que siempre duraron más de 1 hora y 16 minutos. Se observó una mayor implicación, incluso emocional, y una preocupación por el tema por parte de las mujeres entrevistadas al hablar sobre la asistencia prestada.

A partir de las respuestas, se recopilaron 11 sugerencias de indicadores, dos de ellos ya utilizados y otros nueve sugeridos, que se presentan en el Cuadro 1.

**Cuadro 1:** Indicadores utilizados y sugeridos para la evaluación de las buenas prácticas de enfermería dirigidas a las mujeres en situación de violencia en la atención primaria de salud – São Paulo, SP, Brasil, 2025.

Indicadores
Número de notificaciones de casos de violencia doméstica
Número de casos de violencia identificados en la Atención Primaria de Salud
Número de referencias realizadas
Número de mujeres atendidas en los servicios de la red referenciadas por la Atención Primaria de Salud
Adhesión a los cuidados, orientaciones
Análisis de la experiencia de la mujer (recepción o dificultades enfrentadas)
Trabajo en equipo (profesionales que atendieron e intervinieron en la situación vivenciada por la mujer)
Análisis de las vulnerabilidades: escolaridad, ingresos, composición y dinámica familiar
Tasa de retorno a los servicios
Tiempo para resolución de la situación que está siendo atendida
Resultado del caso: abandono o alta

## DISCUSIÓN

La caracterización de los participantes muestra que la mayoría de ellos tienen una amplia experiencia en la atención primaria de salud, lo que puede favorecer la identificación y el manejo de situaciones complejas, como la violencia contra la mujer. El predominio del sexo femenino refleja la composición mayoritariamente femenina de la enfermería, frecuentemente asociada a las prácticas de cuidado y acogida. La diversidad en cuanto al tiempo de formación y de actuación sugiere la coexistencia de diferentes generaciones profesionales, lo que puede ampliar las perspectivas sobre el tema. El número de profesionales con especialización*lato sensu*y la presencia de maestros indica una inversión en la formación continua, mientras que la participación en cursos sobre BPE, incluyendo temas relacionados con la violencia contra las mujeres, demuestra la búsqueda de cualificación para el cuidado de las mujeres.

Los/las profesionales entrevistados hicieron suposiciones sobre la realización de una evaluación por parte de las mujeres atendidas, sugiriendo incluso que no la realizaban y que no tenían conocimientos para ello.

El conocimiento de las mujeres implica, además de su nivel de formación, la comprensión del derecho a la salud, la calidad de los servicios y los mecanismos de responsabilidad y eficacia, que pueden verse influidos por el grupo social al que pertenecen estas mujeres y la comunidad en la que se desarrollan^(16)^. Es decir, tanto el impacto de las acciones desarrolladas como la crítica sobre ellas tienen una influencia directa del contexto social en el que se llevan a cabo^(17)^.

Por ejemplo, la evaluación de un servicio puede encontrar interferencias cuando las mujeres atendidas no tienen conocimiento de sus derechos dentro del sistema de salud o de cuestiones como la acogida o la privacidad dentro del servicio. De este modo, la mujer puede no saber que existe la posibilidad de expresar elogios, críticas o sugerencias, o puede entender que lo que se le ha ofrecido era lo posible, y lo posible, en realidad, puede haber estado por debajo de lo recomendado en las políticas o protocolos.

Las herramientas o estrategias que involucran a la comunidad con el objetivo de fortalecer su relación con los profesionales son facilitadoras y promueven la creación de oportunidades para identificar obstáculos o soluciones para mejorar la atención ofrecida dentro de los servicios de salud, lo cual es una parte fundamental para garantizar la responsabilidad social del usuario^(16)^.

Entre los hallazgos, algunas profesionales destacaron la viabilidad de que la mujer evalúe la asistencia que recibe a través de canales institucionales, como la oficina del ombudsman del municipio. Se trata de un dispositivo constitucional democratizador que permite escuchar directamente a la población y tiene como funciones dar voz activa a los individuos, permitiendo críticas, sugerencias o denuncias, posibilitar la ejecución del control social a través de la fiscalización y evaluar la satisfacción del usuario^(18)^. Cabe cuestionar su divulgación e instar a los servicios a difundir la existencia de la oficina del defensor del pueblo y su objetivo, así como el acceso al canal mediante diferentes estrategias, evitando medios que limiten el alcance de determinados grupos sociales, como el acceso exclusivo a través de páginas en línea, redes sociales o teléfono.

Las enfermeras describieron que una forma en la que reconocen la evaluación de la mujer sobre la atención prestada era el acercamiento de la mujer al equipo y la expresión de gratitud. Un estudio dedicado a analizar los indicadores de la atención afirma que la adhesión al plan terapéutico-asistencial y el aumento de la confianza en los profesionales pueden tenerse en cuenta para la evaluación de las BPE^(5)^.

En consonancia con la duda sobre la realización de la evaluación por parte de las mujeres y su experiencia en los servicios, las profesionales sugirieron, y surge en la tercera categoría empírica, la evaluación de la experiencia de las mujeres al recibir la atención. La experiencia antes, durante y después de la atención, además de cómo y cuándo la persona utiliza y necesita el servicio de salud, son aspectos que inciden directamente en la satisfacción. A pesar de la influencia que la experiencia tiene sobre la satisfacción, es importante destacar que la satisfacción es un concepto relativo, influenciado por aspectos como la expectativa y la percepción de la persona sobre los servicios recibidos y en relación con los profesionales que la atendieron^(19)^. La persona puede estar satisfecha incluso sin haber tenido una buena experiencia, lo que puede llevarla a no volver al servicio^(20)^.

La experiencia del usuario recibió la atención del Ministerio de Salud de Brasil en una Ordenanza que establece indicadores para el pago por desempeño de la APS, para los años 2021 y 2022, incluyendo indicadores de evaluación de la calidad de la atención y la experiencia del paciente^(21)^. El manual de Autoevaluación para Mejorar el Acceso y la Calidad (AMAQ en la sigla en portugués), de carácter reflexivo y problemático, incluye en la evaluación del proceso de prestación de servicios de salud, además de a los profesionales y gestores, al usuario del servicio en lo que se refiere a la participación, el control social y la satisfacción^(22)^. A pesar de ello, en la presente investigación no se citaron estrategias o elementos de evaluación propuestos en los documentos mencionados.

En cuanto a la realización de evaluaciones gerenciales de las BPE, surgieron dudas sobre su ejecución formalizada. Esto corrobora lo que la literatura evidencia como una laguna en la implementación de evaluaciones sobre las prácticas de los profesionales de la salud. La evaluación sigue siendo un reto para la mayoría de los profesionales que trabajan en este ámbito, dada la complejidad del objeto^(5)^.

Se destacó la realización de autoevaluaciones, de forma empírica, por parte de los profesionales. Dado que la autoevaluación citada no cumple con los preceptos rectores, se convierte en un proceso reflexivo del profesional sobre su actuación. Es importante que la acción de autoevaluación se formalice y se oriente, para que los profesionales se perciban dentro de un proceso seguro de cambio que implica movimiento, escucha y consideración de posibilidades que contribuirán a la planificación de acciones futuras, señalando nuevos razonamientos y nuevas soluciones^(23)^.

Una enfermera afirmó que hay una discusión anual sobre la atención a las mujeres en situación de violencia con la gestión municipal de la APS. El hecho de que esta acción no haya sido mencionada por los demás puede estar relacionado con el hecho de que no la comprenden como una estrategia integral de las BPE. Los gestores de los servicios de salud no siempre tienen la formación técnica adecuada o una comprensión integral de las prácticas implementadas por los equipos. Es necesario brindar apoyo técnico a los gestores para que asuman la función y orienten las estrategias de intervención y evaluación sobre las necesidades de los grupos presentes en el territorio^(24)^.

Es fundamental que los gestores comprendan que las estrategias de evaluación de las BPE requieren conocimientos sobre el campo de la salud colectiva y que la asistencia se lleva a cabo en el territorio geopolítico de producción y reproducción social, ya que el trabajo en salud tiene como objetivo la transformación de los perfiles epidemiológicos de la población^(1)^.

Se citaron como indicadores ya utilizados: el número de notificaciones de casos de violencia doméstica e indicadores de vulnerabilidad.

La señalización de la importancia de la notificación de los casos contradice los estudios que discuten la subnotificación de la violencia contra las mujeres^(25,26)^. La notificación se considera una acción importante para el seguimiento de los casos y puede contribuir a planificar, implementar y evaluar las políticas públicas de lucha contra la violencia^(25)^.

El análisis de las vulnerabilidades fue descrito por una entrevistada, que hizo referencia al uso de escalas con este objetivo. Un indicador de vulnerabilidad no es necesariamente un indicador de BPE, sin embargo, identificar la vulnerabilidad de las mujeres de un territorio es fundamental para establecer acciones para enfrentar y superar la violencia. Las desigualdades sociales, incluida la desigualdad de poder entre los géneros, impactan negativamente en las relaciones afectivas e interpersonales, en el acceso a los derechos y en el proceso de reproducción social^(27)^.

Según lo citado por la TIPESC, los indicadores de vulnerabilidad pueden apoyar la evaluación de la asistencia que considera las condiciones de vida y de trabajo de los individuos y los grupos sociales, pudiendo orientar las acciones de cuidado de manera equitativa^(6)^. Además, permiten al Estado reconocer las fragilidades que experimenta la población y contribuir al debate y la implementación de políticas de enfrentamiento^(28)^.

Los indicadores enumerados en esta investigación presentan una fuerte convergencia con las categorías de indicadores de BPE descritas en un artículo que, aunque no se dedica al estudio de las BPE en mujeres en situación de violencia, investiga los indicadores de BPE dirigidos a grupos vulnerables^(6)^. Los resultados evidencian un enfoque integral y sensible a las vulnerabilidades sociales. Por ejemplo, indicadores como «análisis de las vulnerabilidades: escolaridad, ingresos, composición y dinámica familiar» dialogan directamente con la categoría «uso de características sociodemográficas para estimar riesgos o vulnerabilidades». Por su parte, el «análisis de la experiencia de la mujer», la «adhesión a los cuidados» y la «tasa de retorno al servicio o servicios» reflejan aspectos del proceso asistencial muy destacados. Esta articulación refuerza la importancia de las prácticas de enfermería que no solo sean clínicas, sino también sociales, acogedoras e interprofesionales, especialmente en la atención a mujeres en situación de violencia.

El análisis de los indicadores enumerados por las participantes en cuanto a su pertinencia permitió separarlos en tres grupos:

a) Evaluación de las vulnerabilidades de las mujeres a partir de características sociodemográficas, como: ingresos, nivel educativo, situación familiar, lugar de residencia, empleo, entre otras;
b) Evaluación de la construcción de la confianza y el vínculo con el servicio: análisis de la experiencia de la mujer en el servicio y tasa de retorno;
c) Evaluación del proceso de atención: número de notificaciones, número de casos identificados en la APS, número de derivaciones, adhesión a las orientaciones, trabajo en equipo, tiempo de resolución y desenlace del caso.

A pesar de la mención del desenlace como indicador, con la posibilidad de dar lugar al abandono o al alta, no se encontró ninguna referencia a su validación y aplicación en el servicio. Se entiende que la recolección y el análisis de este indicador se realizarían a largo plazo, ya que está directamente influenciado por el período de seguimiento y está relacionado con la adherencia de la mujer a las orientaciones y derivaciones realizadas en la unidad de salud y en la red.

Como evaluación del proceso de atención, se sugirieron indicadores de carácter cualitativo y cuantitativo. El enfoque cuantitativo es importante para conocer la cobertura, la concentración y la eficiencia de los programas, las acciones y las intervenciones, así como para evaluar objetivos específicos. Los enfoques cuantitativos y cualitativos no son antagónicos, sino complementarios, de modo que la combinación de métodos puede contribuir a reconocer la realidad que viven muchas comunidades al acceder a un servicio de salud^(29)^.

A pesar de la complementariedad de ambas vertientes, el análisis de los indicadores sugeridos y categorizados como evaluación del proceso de atención no sugiere una orientación metodológica definida. De este modo, tanto el proceso como su evaluación pueden beneficiarse de la construcción de instrumentos con una orientación teórico-metodológica definida, como la TIPESC, para permitir al enfermero ejercer una crítica preocupada por el cambio del modo actual de organización de la sociedad, las políticas de lucha contra la violencia y las prácticas de intervención en el fenómeno^(30)^.

Bajo la orientación teórico-metodológica de la TIPESC, conviene la composición de un instrumento con el objetivo de reinterpretar la realidad objetiva, antes captada y objeto de intervenciones, descritas en la categoría Buenas Prácticas de Enfermería para mujeres en situación de violencia doméstica: vínculo y acogida como protagonistas de la asistencia en la Atención Primaria de Salud. De esta manera, los indicadores sugeridos por los profesionales pueden componer en el futuro un instrumento que permita la evaluación del producto y del proceso.

Esta investigación presentó como limitaciones su desarrollo durante la pandemia de COVID-19, lo que exigió la realización de entrevistas por teléfono, en sustitución de las entrevistas presenciales. A pesar de ello, la disposición de los profesionales a participar permitió recopilar datos en gran cantidad y calidad.

Se cree que los resultados obtenidos favorecen el desarrollo de nuevas investigaciones que aborden la evaluación de la BPE desde la perspectiva de la Enfermería en Salud Colectiva, así como el desarrollo de instrumentos de evaluación orientados a las prácticas de atención a las mujeres en situación de violencia. Cabe destacar que los resultados tienen el potencial de formar parte de la etapa de construcción del proyecto de intervención en la realidad objetiva, la tercera etapa de la TIPESC.

## CONCLUSIÓN

Se identificaron dos indicadores que se utilizan actualmente para evaluar los BPE en mujeres en situación de violencia, a saber, la notificación de la violencia y la escala de vulnerabilidad. Los indicadores mencionados son importantes, pero insuficientes cuando se analizan de forma aislada. La ausencia de una metodología estructurada y orientada por los preceptos de la Salud Pública limita la capacidad de explorar e interpretar cuestiones sociales importantes que se inscriben en el contexto de vida y trabajo de las mujeres atendidas, lo que hace que la evaluación de las BPE siga siendo un reto.

El estudio presenta un escenario que, aunque impregnado de prácticas asistenciales orientadas por un protocolo local y políticas públicas estructuradas, aún carece de herramientas para realizar evaluaciones formales y específicas, tanto para las usuarias de los servicios como para los profesionales y los gestores.

A pesar de ello, la propuesta de indicadores por parte de los propios profesionales revela el potencial para la construcción colaborativa de instrumentos de evaluación más sensibles a la realidad de los servicios y a las especificidades de las usuarias. Además, la orientación teórico-metodológica de la TIPESC demostró una gran posibilidad de articulación entre la práctica y la crítica social, permitiendo reinterpretar realidades y construir intervenciones eficaces.

## Data Availability

Todo el conjunto de datos que apoya los resultados de este estudio fue publicado en el propio artículo.
